# Supplementary material for: In silico analysis of local RNA secondary structure in influenza virus A, B and C finds evidence of widespread ordered stability but little evidence of significant covariation
Source: Sci Rep. 2022 Jan 10;12:310. doi: 10.1038/s41598-021-03767-x (PMC8748542; doi:10.1038/s41598-021-03767-x)
Supplement: Supplementary file 1 — Supplementary Information. [file 41598_2021_3767_MOESM1_ESM.zip › Supplemental Files/Supplemental File 3.zip!/Supplemental File 3/CM-CAll-rev/pdf/ICV_seg3_1_1.fold.R2R.sto.pdf]

ICV_seg3_1_1

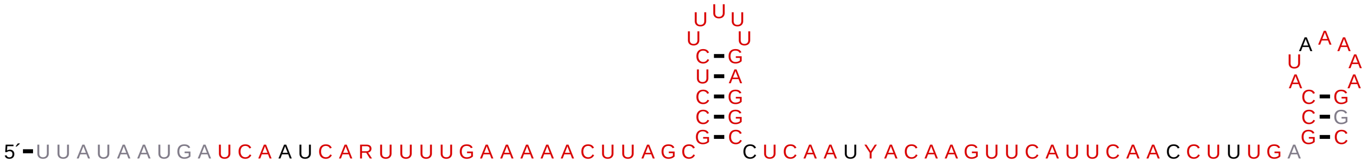
5´ U U A U A A U G A U C A A U C A R U U U U G A A A A A C U U A G C
G C C U C
U U U U U
G A G G C
C U C A A U Y A C A A G U C A U U C A A C C U U U G A
G C C
A U A A A A A
G G C
